# Genes involved in barley yellow dwarf virus resistance of maize

**DOI:** 10.1007/s00122-014-2400-1

**Published:** 2014-09-28

**Authors:** Frederike Horn, Antje Habekuß, Benjamin Stich

**Affiliations:** 1 Max Planck Institute for Plant Breeding Research, Carl-von-Linné-Weg 10, 50829 Cologne, Germany; 2 Julius Kuehn-Institute, Erwin-Baur-Str. 27, 06484 Quedlinburg, Germany

## Abstract

***Key message*:**

**The results of our study suggest that genes involved in general resistance mechanisms of plants contribute to variation of BYDV resistance in maize.**

**Abstract:**

With increasing winter temperatures in Europe, *Barley yellow dwarf virus* (BYDV) is expected to become a prominent problem in maize cultivation. Breeding for resistance is the best strategy to control the disease and break the transmission cycle of the virus. The objectives of our study were (1) to determine genetic variation with respect to BYDV resistance in a broad germplasm set and (2) to identify single nucleotide polymorphism (SNP) markers linked to genes that are involved in BYDV resistance. An association mapping population with 267 genotypes representing the world’s maize gene pool was grown in the greenhouse. Plants were inoculated with BYDV-PAV using viruliferous *Rhopalosiphum padi*. In the association mapping population, we observed considerable genotypic variance for the trait virus extinction as measured by double antibody sandwich enzyme-linked immunosorbent assay (DAS-ELISA) and the infection rate. In a genome-wide association study, we observed three SNPs significantly [false discovery rate (FDR) = 0.05] associated with the virus extinction on chromosome 10 explaining together 25 % of the phenotypic variance and five SNPs for the infection rate on chromosomes 4 and 10 explaining together 33 % of the phenotypic variance. The SNPs significantly associated with BYDV resistance can be used in marker assisted selection and will accelerate the breeding process for the development of BYDV resistant maize genotypes. Furthermore, these SNPs were located within genes which were in other organisms described to play a role in general resistance mechanisms. This suggests that these genes contribute to variation of BYDV resistance in maize.

**Electronic supplementary material:**

The online version of this article (doi:10.1007/s00122-014-2400-1) contains supplementary material, which is available to authorized users.

## Introduction

*Barley yellow dwarf virus* (BYDV) is a group of plant viruses which are of high commercial relevance in small grain cereals (Miller and Rasochová 1977). The virus is exclusively transmitted by a number of aphid species (Rochow 1960). During autumn, the aphids transmit the virus to the autumn-sown cereals by sucking plant sap. In spring, after the sexual reproduction and overwintering, the aphids migrate to autumn-sown cereals, where they acquire the virus and transmit it to the spring cereals as well as to maize in early summer (Henry and Dedryver 1989). Maize represents an important component in the transmission cycle of this virus during summer (Comas et al. 1993) until autumn-sown cereals start growing again.

Fifteen years ago, BYDV has been detected to be a problem for maize cultivation in southern Europe (Beuve et al. 1999; Coceano and Peressini 1989; Comas et al. 1993; Haak et al. 1999; Loi et al. 1986). Bebber et al. (2013) reported that pests and pathogens move further North due to increasing winter temperatures. This is also expected for BYDV which is reported to be also a problem for maize cultivation in Germany (Grüntzig et al. 1997). The reason for the movement northwards is that mild winters allow aphids to overwinter in big populations and infect maize plants at early development stages, when plants are highly susceptible to virus infections (Harrington et al. 2007; Leather 1980).

The control of the virus itself is not possible and also the control of BYDV-transmitting aphids by insecticides in the field is expensive and environmentally unfriendly. Therefore, resistant varieties are the most effective solution to control the virus (Ordon et al. 2004). In breeding, the phenotypic selection on symptoms for BYDV resistance is not promising, as tolerance mechanisms have been reported. This means that the plants do not show any symptoms, but BYDV can multiply and spread in the plant (Grüntzig et al. 1997; Horn et al. 2013). To break the transmission cycle of BYDV, resistant maize in which the virus cannot replicate is of high importance and could contribute to a reduction of the BYDV problems in small grain cereals.

The labor-, cost- and time-consuming method to inoculate the plants in the field and the analysis of virus extinction in the plant by double antibody sandwich enzyme-linked immunosorbent assay (DAS-ELISA) (Clark and Adams 1977) is often too complicated or even impossible to include in practical breeding programs. To accelerate the selection of resistant genotypes in the breeding process, molecular markers linked to resistance genes for marker-assisted selection are an important avail. Previous studies described genetic material showing not only tolerance but also resistance to BYDV (Grüntzig and Fuchs 2000; Horn et al. 2013; Loi et al. 1986). But, to the best of our knowledge, nothing is known about the mechanisms and the inheritance of BYDV resistance and tolerance in maize.

Different resistance mechanisms against viruses in plants have been reported. Many plant species defend themselves passively by strengthened cell walls (Goldbach et al. 2003). *R* genes that are involved in the resistance to viruses belong to the largest class of *R* genes with a nucleotide-binding site plus leucine-rich repeat (NB-LRR) (Goldbach et al. 2003). For *Sugar cane mosaic virus* (SCMV), two major resistance genes have been mapped to chromosomes 6 (*Scm1*) and 3 (*Scm2*) (Melchinger et al. 1998) as well as three minor genes to chromosome 10 (Xia et al. 1999; Zhang et al. 2003). In SCMV resistant maize genotypes, the virus spread was slower than in susceptible plants which leads to the assumption that in resistant genotypes the virus spread through the leaf vascular system is inhibited (Quint 2003). Zambrano et al. (2014) identified QTL on the chromosomes 1, 2, 3, 6 and 10 contributing to resistance against six virus diseases. Furthermore, Jones et al. (2011) and McMullen and Simcox (1995) identified the locus *wsm3* on chromosome 10 which explains variation of *Wheat strike mosaic virus* (WSMV) resistance. Jones et al. (2004) described the locus *mcd2* to contribute to resistance against *Maize chlorotic dwarf virus* (MCDV). BYDV resistance is described to be quantitatively inherited in barley (Riedel et al. 2011; Toojinda et al. 2000) and wheat (Ayala et al. 2002). We observed in an earlier study (Horn et al. 2013) that the values of the trait virus extinction (EX) estimated by DAS-ELISA were normally distributed in the examined germplasm which suggests a quantitative inheritance. In maize and other species it was reported that translation factors such as eucariotic initiation factor *eIF4E* play a role in resistance strategies against plant viruses (Kang et al. 2005; Zambrano et al. 2014; Zhang et al. 2006). To our knowledge, however, there are no resistance genes against BYDV described in maize yet. A promising approach to identify markers genetically linked to the trait of interest is genome-wide association mapping. This method has the advantage that a large number of alleles per locus can be surveyed simultaneously, and because historical recombinations can be used, the mapping resolution is higher compared to classical QTL mapping (Flint-Garcia et al. 2005).

The objectives of this study were to (1) determine genetic variation with respect to BYDV resistance in a broad germplasm set of maize and (2) to identify single nucleotide polymorphism (SNP) markers linked to genes that are involved in BYDV resistance.

## Materials and methods

In our study, we used 267 inbred lines from a set of 302 genotypes which represent the world wide diversity of maize breeding programs (Flint-Garcia et al. 2005). The set comprised stiff stalk (SS), non stiff stalk (NSS) as well as tropical and subtropical inbred lines (TS). Furthermore, it includes some popcorn and sweetcorn inbreds. The experiment was carried out in the greenhouse with two replicates, each with ten plants per genotype. Each plant was grown in a pot with 300 ml of “Profi Substrat” soil (Einheits Erde^®^ Classic) with a day–night temperature regime of 16 h/24 $^\circ$C and 8 h/22 $^\circ$C. The experimental design was an $\alpha$-lattice design.

Viruliferous *Rhopalosiphum padi* were multiplied for three weeks in a growth chamber at 20$^\circ$C on *Triticum aestivum* cultivar “Tuareg” infected with BYDV-PAV. Ten days after sowing, in the 2–3 leaf stage, the maize plants were inoculated by placing a piece of wheat leaf with about 5–10 aphids in the leaf axil of each plant. To prevent an escape of the aphids, all plants of one replicate were covered with plant protection fleece [*Climatex*] (17 g/qm). One week after inoculation, the plant protection fleece was removed and the plants were sprayed with the insecticide “Lizetan Plus” (Bayer) to stop inoculation.

To analyze the BYDV content, six weeks after the inoculation start, plant material from the sixth leaf was collected separately for each plant and each leaf was analyzed by DAS-ELISA according to Clark and Adams (1977) using the polyclonal antisera (BYDV-PAV) from the Julius Kuehn Institute (JKI). EX was estimated at 405 nm on a microtiter plate reader (Opsys MR, Egelsbach, Germany) 1 h after the incubation of the enzyme substrate. In Grüntzig and Fuchs (2000) and Loi et al. (1986), the lines Ky226 and FAP1360A were described to be resistant against BYDV. In a previous study by Horn et al. (2013) these lines showed EX values of 0.30 and 0.26. In contrast, the inbreds W64A and P092 which were described to be susceptible (Loi et al. 1986) showed values of 0.83 and 1.02. Therefore, we designated plants with EX <0.5 as resistant The infection rate (IR) was calculated as the percentage of plants of one plot with EX ≥0.5 (Horn et al. 2013).

### Mutant screening

**Fig. 1:**
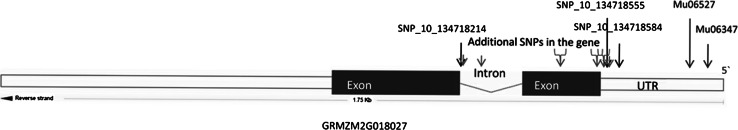
Gene model of the gene GRMZM2G018027 from maizesequence.org with the position of the three significantly (FDR = 0.05) associated SNPs, the SNPs further available in this gene and the two Mu-mutations

The two F3 sib-pollinated families, Mu06347 and Mu06527, from the Maize Genetics Cooperation Stock Center with a 9 bp insertion in the 5$^{\prime }$UTR (untranslated region) of the gene GRMZM2G018027 (Fig. 1) were examined together with the inbred line W22 which is the wild type background for the mutants. The plants were grown in pots with a volume of 300 ml, in “Profi Substrat” soil with a day–night temperature regime of 16 h/26 $^\circ$C and 8 h/22 $^\circ$C in a climate chamber as described above for the association mapping population. A total of 15 seeds of each mutant genotype and 48 seeds of W22 were randomized in a complete block design with four replicates.

Two replicates were inoculated with BYDV and two served as a non-inoculated control. The inoculation and the analyses of EX were performed as described above. To test whether the insertions are present, we designed the following polymerase chain reaction (PCR) primers: forward primer (5′-GCGAAACCGAGTAGGTGGA-3′) and reverse primer (5′-GAGGGAATGGAGGAAGGAAG-3′) for the 5′UTR of the gene using the software Primer3 (Rozen and Skaletsky 2000). The PCR product of this region was sequenced for the mutant inbreds and the wild type by the DNA core facility of the Max Planck Institute for Plant Breeding Research on Applied Biosystems (Weiterstadt, Germany) Abi 3730XL sequencers using BigDye-terminator v3.1 chemistry. Premixed reagents originated from Applied Biosystems (Foster City, CA, USA) as described by Benke and Stich (2011).

### Statistical analyses for the association mapping population

#### Phenotypic data analyses

We used the following mixed model to analyze the data of all collected traits:

1 $$\begin{aligned} Y_\text {ijk} = \mu + r_\text {i} + g_\text {j} + rb_\text {ik} + e_\text {ijk}, \end{aligned}$$

where $Y_\text {ijk}$ was the phenotypic observation for the *i*th replicate for the *j*th genotype in the *k*th incomplete block, $\mu$ the general mean, $r_{i}$ the effect of the *i*th replication, $g_{j}$ the effect of the *j*th genotype, $b_{k}$ the effect of the *k*th block, and $e_\text {ijk}$ the residual. We regarded *r* and *g* as fixed effects, whereas the interaction effect rb was regarded as random. With this model, we calculated the adjusted means for each genotype and, with *g* as a random effect, the genotypic $\sigma _{{\text{g}}}^{2}$ and error $\sigma _{{\text{e}}}^{2}$ variance. Broad-sense heritability ($H^{2}$) was calculated based on the formula

2 $$\begin{aligned} {\mathrm{{\it H}}}^{2} = \frac{\sigma ^{2}_{\mathrm{g}}}{\sigma ^{2}_{\mathrm{g}} + \dfrac{\sigma ^{2}_{\mathrm{e}}}{n}}, \end{aligned}$$

where *n* was the number of replications. All mixed model analyses were performed using the software ASReml (Gilmour et al. 2006).

For the comparison of the mutant genotypes with their wild type W22, *t*-tests were performed for the traits EX and IR using the statistical software R (R Core Development Team 2011).

#### Association mapping analyses

For the association mapping population, information of about 437,650 SNP markers is available from http://www.panzea.org. After the exclusion of markers with >20 % missing data across all inbreds and markers with an allele frequency <2.5 %, 286,838 markers remained for the following analyses. The genome-wide association analyses of the traits, EX and IR, were performed for each of the 286,838 SNP markers with the R package EMMA (Kang et al. 2008) using the QK model:

3 $$\begin{aligned} M_{jp} = \mu + m_{p} + \sum \limits _{u=1}^{z}Q_{ju}v_{u} + g_{j}^{*} + e_{jp}, \end{aligned}$$

where $M_{jp}$ is the adjusted entry mean of the genotype *j* in the *p*th allele, $m_{p}$ the effect of allele *p*, $v_{u}$ is the effect of the *u*th column of the population structure matrix *Q*, $g_{j}^{*}$ is the residual genetic effect of the *j*th entry and $e_{jp}$ the residual. The variance of the random effect $g^{*}$ = {*g* $^{*} _\text {1},\ldots ,g^{*}_\text {267}$} was assumed to be Var($g^{*}$) = 2$K\sigma ^{2}_{g^{*}}$, where *K* is a 267 $\times$ 267 matrix of kinship coefficients that define the degree of genetic covariance between all pairs of entries, and $\sigma ^{2}_{g^{*}}$ is the genetic variance estimated by REML (Stich et al. 2008).

The population structure matrix *Q* from Flint-Garcia et al. (2005) was used in our study. As significance threshold we chose the false discovery rate (FDR) = 0.05 (Hochberg and Benjamini 1995).

To be able to identify genes which are linked to the significantly associated SNPs, we considered the extent of linkage disequilibrium (LD), which extends 2,000 bp in maize (Remington et al. 2001), as threshold. All genes within the range of 2,000 bp around the physical position of the significantly associated SNPs were extracted from the filtered gene set of the maize genome sequence ZmB73_5b_FGS.

## Results

**Fig. 2:**
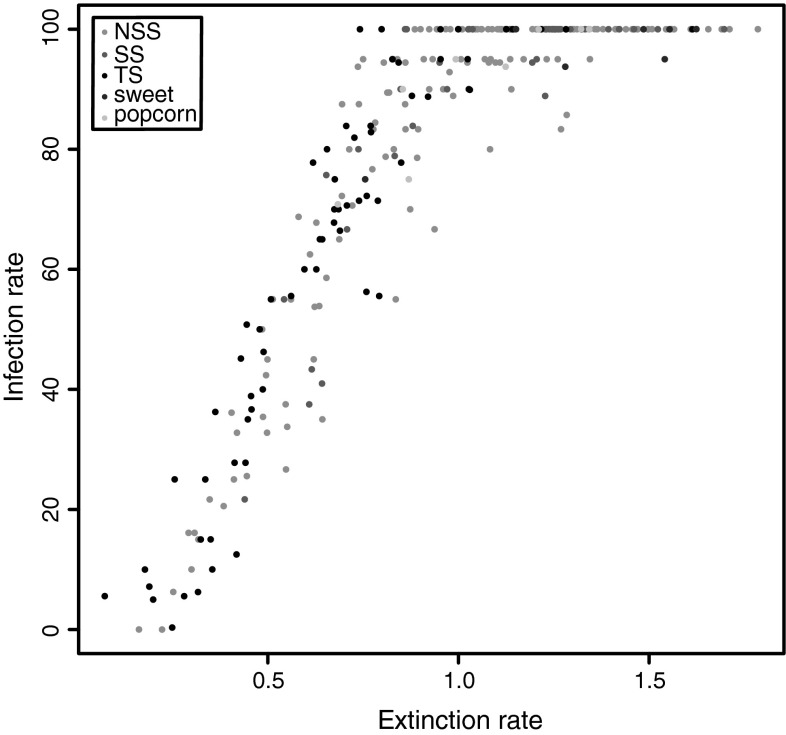
Covariation of the traits virus extinction (EX) and infection rate (IR) in the association mapping population of 267 genotypes. *NSS* are the non stiff stalk accessions, *SS* the stiff stalk accessions, *TS* the tropical and subtropical lines, sweet the sweet corn accessions, and popcorn the popcorn accessions

The $H^{2}$ for the traits, EX and IR, were high with 0.84 and 0.83 (Table 1) in the association mapping population. With a value of $\rho$ = 0.86, the two traits were highly correlated (Fig. 2). For the traits EX and IR, we observed a broad variation in the entire population as well as in all subgroups, with EX values ranging from 0.07 to 1.79 and with trait values ranging from 0 to 100 % for IR (Fig. 2 and Online Resource 1 and 2). The phenotypic variation explained by the population structure matrix *Q* was 7.0 % for the trait EX and 4.7 % for the trait IR.

**Table 1:** Analysis of variance for the traits virus extinction (EX) and infection rate (IR) in the association mapping population

Parameter	Traits	
	EX	IR
Broad-sense heritability ($H^{2}$)	0.84	0.83
Genotypic variance	0.12	691.35
Mean	0.93	78.20
Standard deviation of the mean	0.37	29.00
Range	0.06–1.79	0.00–100.00

**Fig. 3:**
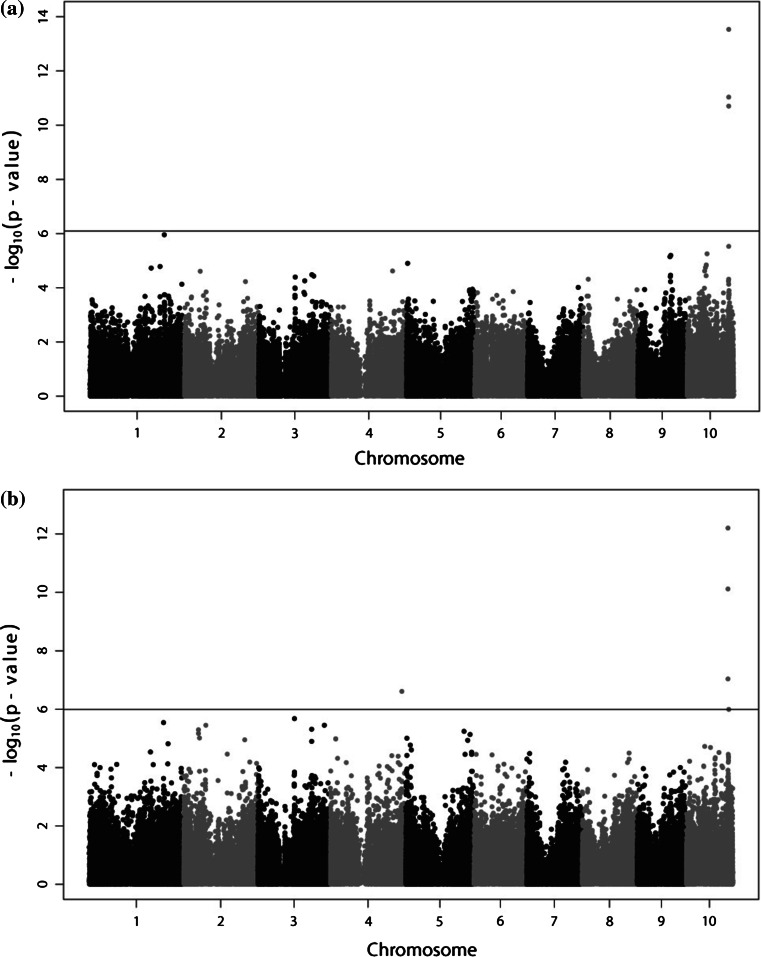
Manhattan plots from the genome-wide association study showing significantly (FDR = 0.05) associated SNPs with the traits: **a** virus extinction (EX) and **b** infection rate (IR)

On chromosome 10, we identified three SNPs that were significantly (FDR = 0.05) associated with the trait EX (Table 2; Fig. 3a). Three SNPs explained between 16 and 21 % of the phenotypic variance each, with a simultaneous fit of the three SNPs explaining 25 % of the phenotypic variance of the trait EX. All three SNPs were located within the non-coding region of gene GRMZM2G018027. One is located in the intron and two in the 5$^{\prime }$UTR (Fig. 1).

**Table 2:** Single nucleotide polymorphisms (SNPs) significantly (FDR = 0.05) associated with the traits virus extinction (EX) and infection rate (IR) for the association mapping population with 267 genotypes

Trait	Marker locus	Gene	Chromosome	Position in bp	*P* value	Allele 1/2	Allele effect 1–2	Proportion of explained $\sigma _{{\text{g}}}^{2}$ (%)
EX	S10_134718214	GRMZM2G018027	10	134,718,214	2.95e−14	A/G	0.38	20.71
	S10_134718555	GRMZM2G018027	10	134,718,555	9.25e−12	C/T	0.31	16.74
	S10_134718584	GRMZM2G018027	10	134,718,584	1.98e−11	A/G	0.31	16.10
	Simultaneous fit							24.86
IR	S10_134718214	GRMZM2G018027	10	134,718,214	6.23e−13	A/G	28.50	18.14
	S10_134718555	GRMZM2G018027	10	134,718,555	9.31e−08	C/T	19.93	10.67
	S10_134718584	GRMZM2G018027	10	134,718,584	7.59e−11	A/G	24.37	15.20
	S10_136961730	GRMZM5G856306	10	136,961,730	1.02e−06	C/T	19.33	09.30
	S4_228661279	GRMZM2G121790	4	228,661,279	2.47e−07	C/A	28.69	10.00
	Simultaneous fit							33.36

These three SNPs were also significantly associated with the trait IR and explained between 11 and 18 % of the phenotypic variance each (Table 2; Fig. 3b). Furthermore, an additional SNP on chromosome 10 (FDR = 0.05) within the gene model GRMZM5G856306 explained 9 % of the phenotypic variance. On chromosome 4, a fifth significantly associated SNP, which explained individually 10 % of the phenotypic variance, was identified within the gene model GRMZM2G121790. All five significant SNP-IR associations explained together in a simultaneous fit 33.36 % of the phenotypic variance of IR.

In the mutant genotype experiment, the mean EX values differed between the mutant genotypes and the wild type genotype. The EX value of the mutant genotype Mu06347 was with 0.47 two times higher compared to the wild type W22 (0.23) and Mu06527 even three times higher (0.70). The same was true for IR with values from 37.5 % for the mutant genotype Mu06347 and 50.5 % for Mu06527 compared to W22 with 15.7 %. Nevertheless, the observed differences were not significantly different from 0 ($\alpha$ = 0.05).

## Discussion

### Phenotypic trait variation in the association mapping population

We observed a broad and continuous variation in the traits EX and IR in the association mapping population (Fig. 2). Such a distribution was also shown in a previous study (Horn et al. 2013) for segregating populations. This observation together with the high $H^{2}$ (Table 1) indicates that an improvement of BYDV resistance in maize is possible by breeding for a low virus extinction. In this association mapping population, the genetic variance was even broader compared to the segregating populations studied by Horn et al. (2013). This high genetic variance in BYDV resistance could be due to the different genetic backgrounds of inbred lines from all over the world. We observed a high correlation of the traits EX and IR (Fig. 2). This was expected because IR is calculated from EX.

Phenotypic diversity for IR and EX was observed in the entire population and also within the five population subgroups. Furthermore, there was no clear clustering for the studied traits between the subgroups. The lack of sub-population effect was also supported by the observation that only 7 % of the phenotypic variance in the trait EX and 4 % in the trait IR can be explained by population structure. The reason for this finding could be the absence of divergent selection pressure in the different subgroups for these traits. Therefore, association mapping was performed across the whole association mapping population.

### Genes significantly associated with BYDV resistance in maize and their presumable function

The SNPs significantly (FDR = 0.05) associated with the two traits EX and IR were all located in the sequences of previously described genes. The genes which are physically close to significantly associated SNPs are not necessarily the causal genes for the observed trait variation. Nevertheless, we found evidence that suggests such a functional relationship in physical proximity to the associated SNPs. The three SNPs on chromosome 10 which are significantly associated with EX and IR are located in the sequence of the gene GRMZM2G018027. The best hit for this gene in *Arabidopsis thaliana* is the gene *OXS3* which was described to be expressed during the oxidative stress reaction (Blanvillain et al. 2009) and enhanced the tolerance to heavy metals like cadmium and other oxidizing chemicals. Wang and Culver (2012) reported that resistance to *Tobacco mosaic virus* and cadmium treatment is associated. The reason could be that *OXS3* produces H$_{2}$O$_{2}$ which leads to the induction of a cadmium-induced glycine-rich protein which supports callose deposition within the plasmodesmata and vascular cell walls (Ueki and Citovsky 2005) which in turn reduces the virus spread in the plant (Wang and Culver 2012). This means that it is likely that *OXS3* improves virus resistance by the production of H$_{2}$O$_{2}$ (Wang and Culver 2012). The same could be true for BYDV resistance in maize.

Besides the callose deposition, H$_{2}$O$_{2}$ was also associated with other resistance mechanisms. Doke et al. (1996) reported that oxidative burst plays an important role as an emergency signal during pathogen attacks to activate the defense, which is in many cases a hypersensitive reaction but at times also a protective agent (Lamb and Dixon 1997) or the cross-linking of cell wall proteins (Levine et al. 1994).

Tao et al. (2013) found a candidate gene for the SCMV resistance gene *Scmv1* on chromosome 6 in maize. This candidate gene *Zmtrx-h* encodes a putative 119-residue protein that is highly similar to h-Type Thioredoxin. Thioredoxin proteins regulate redox pathways and induce systemic acquired resistance (SAR). Therefore, its overexpression leads to resistance to *Tobacco mosaic virus* and *Cucumber mosaic virus* and also improved tobacco resistance to oxidative stress (Sun et al. 2010). This finding leads to the assumption that the production of H$_{2}$O$_{2}$ by *OXS3* may also induce SAR which leads to enhanced BYDV resistance in maize.

In the Maize Genetics Cooperation Stock Center (http://maizecoop.cropsci.uiuc.edu/), UniformMu genotypes are available containing an insertion in the gene GRMZM2G018027. To test whether this mutation changes the resistance to BYDV, we compared the mutant genotypes to their wild type inbred W22 with regard to their virus extinction. In the mutant genotypes we found considerably higher EX values compared to the wild type, which suggests that a mutation in the gene could reduce the BYDV resistance. In our study, this difference was, however, not statistically significant. One reason for the non-significance could be that the gene is not the causal gene and therefore, it has no influence on BYDV resistance. Another reason could be, however, that the insertion is located in the 5$^{\prime }$UTR (Fig. 1). Even though we confirmed that the insertion is present in mutant genotypes, it is possible that this insertion does not disturb strongly the binding of the ribosome and the expression of the gene dramatically.

In this study, however, the non-significance can be caused by the low number of tested plants/genotype leading to a high error. Furthermore, in the sib-pollinated F3 plants heterozygote mutants are still present leading to higher variances observed in the mutant genotypes compared to W22 (data not shown). This makes it difficult to observe statistically significant differences. UniformMu genotypes can carry multiple insertions in multiple genes. This can also lead to a high error in the analyses of these genotypes. Therefore, we suggest to study further the effect of *OXS3* on EX and IR in maize.

On chromosome 10 we detected another SNP, which was significantly associated with the trait IR. This SNP is located within the gene GRMZM5G856306 which codes for a zinc finger family protein in *Arabidopsis thaliana* and *Oryza sativa*. It was reported by Uzarowska et al. (2009), Mukhopadhyay et al. (2004), and Yang et al. (2007) that under different types of stresses, including viral and fungal inoculation, zinc finger proteins were induced. Gupta et al. (2012) observed zinc finger domains in the R proteins of nine different crops. Zinc finger proteins are encoded in *Arabidopsis thaliana* by *LSD1* controlling a superoxide pathway and regulating the cell death negatively (Dietrich et al. 1997; Epple et al. 2003).

For the trait IR, we could also detect a significantly associated SNP on chromosome 4. The gene GRMZM2G121790 which is located next to this SNP codes in *Arabidopsis thaliana* for a SHAGGY-related protein kinase. Dogra et al. (2009) found a SHAGGY-like kinase interacting with the tomato leaf curl virus pathogenicity determinant C4 protein. Jonak and Hirt (2002) described that SHAGGY-like kinase proteins are involved in wound responses.

The above described function of the genes allows us to assume that they play a role in resistance mechanisms in their organisms. In our study the significantly associated SNPs to BYDV resistance are located within these genes which are not yet described in maize. However, this could be an indication that also in maize these genes play a role in BYDV resistance mechanisms but this requires further research.

### Virus resistance genes on chromosome 10

In our study, we identified the three strongest associated SNPs with both traits on chromosome 10. In previous studies, several virus resistance loci were described in maize on chromosome 10 (Jones et al. 2004, 2011; McMullen and Simcox 1995; Xia et al. 1999; Zhang et al. 2003; Zambrano et al. 2014). The QTL interval for resistance to *Maize dwarf mosaic virus* (MDMV) (86424631-137500030bp) from the study of Zambrano et al. (2014) covers the three highly associated SNPs in this study which means the same locus could cause resistance to BYDV and MDMV.

Furthermore, the gene *wsm3*, which is associated with resistance to WSMV (McMullen and Simcox 1995; Jones et al. 2011), is located on the long arm of chromosome 10 close to the marker *umc1506*. In this previous study, fewer markers were used compared to the current study and therefore, the markers are about 1,000,000 bp away from our gene of interest. Nevertheless, it could be possible that the same gene confers resistance to several viruses because also the locus *mcd2* for resistance to MCDV (Jones et al. 2004) as well as three minor QTL for SCMV resistance (Xia et al. 1999; Zhang et al. 2003) were identified on chromosome 10 to be associated with *wsm3*.

### The eukaryotic translation initiation factor as a candidate for BYDV resistance?

Kang et al. (2005), Zambrano et al. (2014) and Zhang et al. (2006) reported that translation factors such as eukaryotic initiation factor *eIF4E* play a role in resistance mechanisms against plant viruses in maize and other species. This suggested that eukaryotic initiation factors could also be involved in BYDV resistance in maize. Therefore, we blasted the sequences of different maize eukaryotic initiation factors to the B73 reference genome but for none of the blasted regions we identified significant (FDR = 0.05) associations with the BYDV resistance traits. Therefore, our results suggest that the eukaryotic translation initiation factors are no candidates for BYDV resistance in the examined maize population.

### Application of significantly associated markers in breeding programs

The evaluation of maize genotypes for their BYDV resistance by aphid inoculation and DAS-ELISA analyses is very labor- and cost intensive and therefore, its integration in commercial breeding programs is not promising. Therefore, the development of molecular markers linked to the traits EX and IR is the basic requisite for an effective improvement of BYDV resistance levels. BYDV resistant maize inbreds would contribute to secure a sustainable agriculture. The SNPs which were significantly (FDR = 0.05) associated with the traits EX and IR explained with 24.86 and 33.63 % a considerable proportion of the phenotypic variance. That means their use for marker-assisted selection in breeding maize to improve BYDV resistance is promising.

**Table 3:** DNA sequence 50 bp ahead and 50 bp behind the SNP, flanking the significantly associated SNPs (maizeGDB B73 reference sequence v2)

Gene model	Marker locus	DNA sequence 50 bp ahead the SNP	SNP alleles	DNA sequence 50 bp behind the SNP
GRMZM2G018027	S10_134718214	cgaacgactgcgacttgccggcgaagaacttggacaggcccctcctgcac	A/G	cccaaacaaagcagctgtggcgtcagatcaccgaaccaccgtacccccgc
	S10_134718555	tcctcctgctccttggcgcgcgcgaacaggacgtaggcctccattgcccc	C/T	aactcccaagcagatcacgcggaagaacgaacgaacaaacgatgtaagtg
	S10_134718584	gacgtaggcctccattgcccccaactcccaagcagatcacgcggaagaac	A/G	aacgaacaaacgatgtaagtggaacgatgaagtacgatgctctattgcgg
GRMZM5G856306	S10_136961730	gtgcgggaggccgccgcagagcacctcgaggagcggcaggctgactgggc	C/T	tactcccgccccgtcgtggcgctcgacctgctgtggaacctcgctttcat
GRMZM2G121790	S4_228661279	ctaacacattgtacttcagagcagctaaaaactagattatcttaagtatt	C/A	ttttaggtgctctataaaaaggctagacttacctttgacctttacccttt

From the DNA sequence flanking the SNPs (Table 3), marker assays for the genotyping platforms available in the different breeding companies can be designed. In the next step, breeders could screen with these markers the proprietary genetic material of the different heterotic pools, and see whether the advantageous allele combination is present at the significantly associated SNPs. Afterwards it is important to check the resistance level of this genotype in comparison to the genotype with the disadvantageous allele combination. If this is significantly higher, a selection on the favorable allele combination is promising in breeding populations.

If the allele combination which was found to increase the BYDV resistance in our study is not present in the proprietary genetic material, our results (given in Online Resource Table S1) can be used to identify resistance sources for the different heterotic pools of interest.

## Conclusion

The broad and continuous variation with regard to BYDV resistance in the association mapping population shows that an improvement of BYDV resistance by breeding is possible and provides the condition for the identification of molecular markers linked to BYDV resistance by association mapping. The identified markers are strongly associated with the traits virus extinction and infection rate and explain a considerable part of the phenotypic variation of the studied association mapping population. The results of our study suggest that genes involved in general resistance mechanisms are also involved in BYDV resistance in maize.

### **Author contributions**

FH participated in the design of the experiment, carried out most of the experimental work, performed the statistical analyses, and drafted the manuscript. AH participated in the design of the experiment, experimental work, and drafted the manuscript. BS conceived the study, participated in its design and statistical analyses, and drafted the manuscript. All authors read and approved the final manuscript.

## Electronic supplementary material

Below is the link to the electronic supplementary material.

Supplementary material 1 (pdf 39 KB)
