# Supplementary material for: Genes involved in barley yellow dwarf virus resistance of maize
Source: Theor Appl Genet. 2014 Sep 28;127(12):2575–84. doi: 10.1007/s00122-014-2400-1 (PMC4236618; doi:10.1007/s00122-014-2400-1)
Supplement: Supplementary file 1 — Supplementary material 1 (pdf 39 KB) [file 122_2014_2400_MOESM1_ESM.pdf]

Genes involved in barley yellow dwarf virus resistance of maize

Frederike Horn[1], Antje Habekuß[2] and Benjamin Stich[1*]

[1]Max Planck Institute for Plant Breeding Research, Carl-von-Linné-Weg 10, 50829 Cologne, Germany

[2]Julius Kuehn-Institute, Erwin-Baur-Str. 27, 06484 Quedlinburg, Germany

Theoretical and Applied Genetics

SUPPLEMENTERY

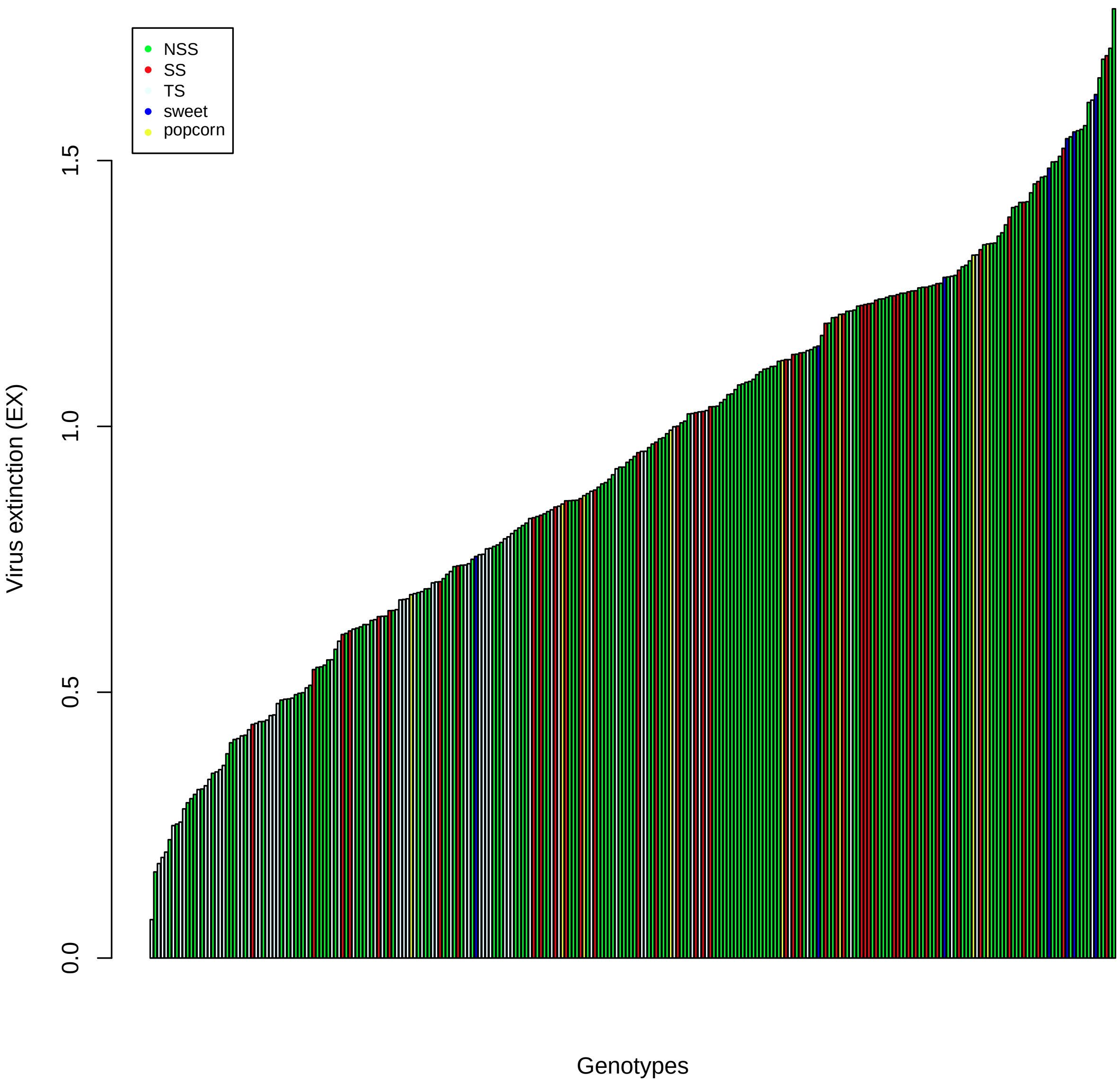

ESM_1 Variation of the trait virus extinction (EX) in the association mapping population of 267 genotypes. NSS are the non stiff stalk accessions, SS are the stiff stalk accessions, TS the tropical and subtropical lines, sweet are the sweet corn accessions and popcorn the popcorn accessions

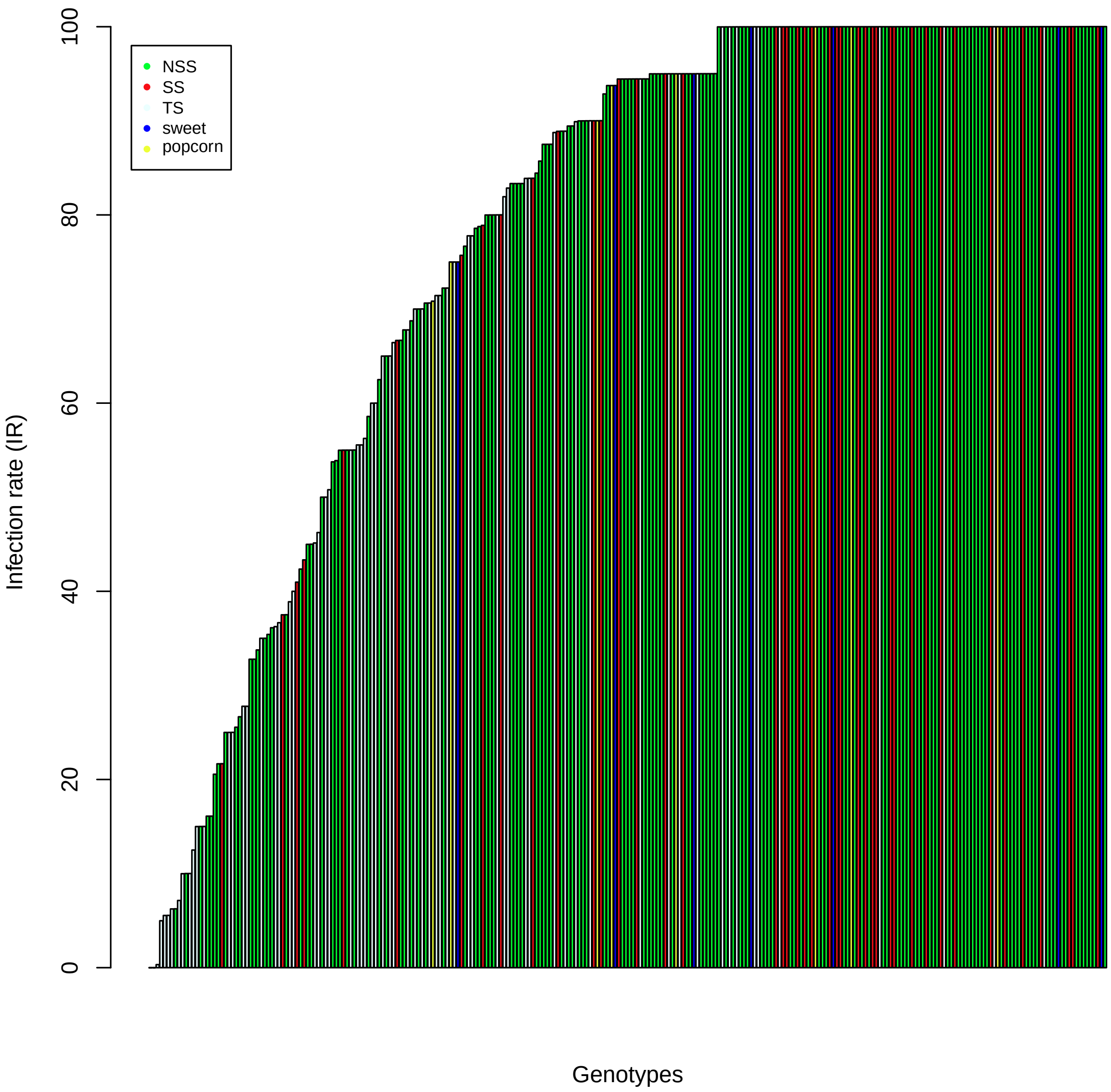

ESM_2 Variation of the trait values observed for infection rate (IR) in the association mapping population of 267 genotypes. NSS are the non stiff stalk accessions, SS are the stiff stalk accessions, TS the tropical and subtropical lines, sweet are the sweet corn accessions and popcorn the popcorn accessions

Table S1. Lines that can be used as resistance source (virus extinction <0.5) with the information about their virus extinction, infection rate and subgroup. Ordered by virus extinction

official ID	Virus extinction	Infection rate	Subgroup
Ames 27123	0.07	5.56	TS
PI 550522	0.16	0.00	NSS
Ames 27089	0.18	9.99	TS
Ames 27087	0.19	7.14	TS
Ames 27076	0.20	4.99	TS
Ames 27086	0.22	0.00	NSS
Ames 27092	0.25	0.33	TS
Ames 27125	0.25	6.25	NSS
Ames 27145	0.26	25.01	TS
Ames 27094	0.28	5.55	TS
PI 550490	0.29	16.10	NSS
Ames 27140	0.30	10.00	NSS
Ames 19328	0.31	16.10	NSS
PI 595544	0.32	6.24	TS
Ames 27112	0.32	15.00	NSS
Ames 27090	0.32	15.00	TS
Ames 27078	0.34	25.00	TS
PI 587148	0.35	21.66	NSS

Table S1 continued.

official ID	Virus extinction	Infection rate	Subgroup
Ames 27071	0.35	15.01	TS
Ames 27084	0.35	10.01	TS
Ames 27102	0.36	36.25	TS
Cize28	0.38	20.56	NSS
PI 587151	0.40	36.12	NSS
PI 558532	0.41	25.00	NSS
Ames 27095	0.41	27.78	TS
Ames 27258	0.42	12.50	TS
Ames 8399	0.42	32.78	NSS
Ames 27091	0.43	45.13	TS
Ames 23405	0.44	21.68	SS
PI 595550	0.44	27.78	TS
Ames 27146	0.44	50.79	TS
Ames 20190	0.45	25.55	NSS
Ames 27129	0.45	35.01	TS
Ames 27148	0.46	38.88	TS
Ames 27072	0.46	36.66	TS
Ames 27126	0.48	50.00	TS
Ames 27080	0.49	50.00	NSS
Ames 27074	0.49	40.00	TS
Ames 19284	0.49	35.42	NSS
Ames 27147	0.49	46.24	TS
